# Negotiating security and resilience: planning conundrums for avian influenza control in Great Britain’s poultry systems

**DOI:** 10.1088/2976-601X/ae8dd4

**Published:** 2026-08-21

**Authors:** Mathew Hennessey, Pablo Alarcon, Paniz Hosseini, James L N Wood, Ivo Syndicus

**Affiliations:** 1 Veterinary Epidemiology, Economics, and Public Health Group, Department of Pathobiology and Population Science, Royal Veterinary College, Hatfield, United Kingdom; 2 Department of Veterinary Medicine, University of Cambridge, Cambridge, United Kingdom; 3 School of Geography and the Environment, University of Oxford, Oxford, United Kingdom

**Keywords:** poultry, avian influenza, biosecurity, resilience

## Abstract

The outbreaks of highly pathogenic avian influenza in Great Britain between 2021 and 2026 represent the most severe period of virus activity recorded in the country, prompting renewed scrutiny of how biosecurity is conceptualised and practised within poultry systems. This paper examines how the structure of poultry sectors, producing meat, eggs, and game birds, shape disease risk and the possibilities for a resilient poultry ecology. Using a production and distribution network approach, we combine a desk-based review of industry, policy, grey, and published literature with qualitative data from 19 in-depth interviews, supplemented by field visits and engagement in stakeholder events. Our analysis shows that highly integrated and geographically concentrated poultry meat and layer systems interact with wild and semi-wild birds in ways which generate distinct challenges. Concentration of production and processing infrastructure, aging housing stock, consumer demand for free-range, and large-scale releases of game birds impact decisions around poultry governance and questions of risk around avian influenza incursion. We identify two cross-cutting ‘entanglements’ that condition avian influenza virus vulnerability: (1) poultry planning, where fragmented and often contested land-use decisions interact with environmental and biodiversity policies to shape exposure to wild and semi-wild ecologies; and (2) the insuring of poultry, where a market failure in private avian influenza insurance and uneven and contested state compensation regimes differentially distribute risk across producers and sectors. We argue that farm-level biosecurity, while necessary, is insufficient to secure Britain’s poultry production under near-endemic avian influenza virus pressure. A more bio-resilient poultry future will require macro-level, multi-sectoral coordination that integrates spatial planning, infrastructure renewal, and equitable risk-sharing mechanisms for disease preparedness.

## 1 Introduction

Since the winter of 2021 and 2022, Great Britain^4^ (GB), like Europe in general, has experienced an unprecedented number of outbreaks of highly pathogenic avian influenza (HPAI) in birds, marking the most sustained and severe period of virus activity in recent times. During the 2021-'22 and 2022-'23 seasons^5^, 158 and 206 infected premises were reported in GB in poultry and captive birds, more than double the number seen in earlier years (APHA 2022, APHA 2026). The large number of cases, which were concentrated in England, led to numerous local restrictions around infected premises, and in November 2021 all of GB was placed under an avian influenza protection zone (AIPZ) as an emergency containment. This resulted in a nationwide ‘flock-down’ (McClaughlin *et al* 2024), a mandatory housing order for commercial and backyard flocks; a measure which lasted for seven months (Defra 2022a).

Unlike previous years, in 2022 the virus did not recede with the onset of warmer months. That summer saw persistent outbreaks, with large numbers of positive cases in wild birds, particularly sea birds in breeding colonies which suffered mass die-offs (APHA 2022). Of the farm outbreaks which occurred in the ‘22 and 23’ season, analysis by the Animal & Plant Heath Agency (APHA) determined that 93% of cases were due to direct and/or indirect contact with wild birds with only a small number of cases occurring due to transmission between infected poultry sites (APHA 2023). This highlights a marked shift from previous views that HPAI incursions were caused by ‘the movement of poultry, poultry products, or infected material from poultry farms’ (The Lancet Infectious Diseases 2006). While the disease challenge abated in ‘23 and 24’, there has been a subsequent resurgence in outbreaks and as of July 2026 there have been 95 confirmed HPAI infected premises (Defra 2026a). These ongoing outbreaks thus present an opportunity to interrogate how poultry ecologies are rendered secure, or insecure, by the wider structures within which they exist.

With each major outbreak of livestock disease, biosecurity re-emerges as a central object of scrutiny and intervention. Biosecurity, broadly conceived, refers to a suite of practices, infrastructures, and regulatory mechanisms aimed at limiting the introduction and spread of infectious pathogens (Enticott 2008). These operate across multiple scales: at the level of individual farms, biosecurity entails on-site measures ranging from infrastructural layout (e.g. buffer zones around housing) to procedural interventions (e.g. disinfectant footbaths, staff clothing protocols) and movement restrictions of animals onto and within farms, all aiming to minimise disease exposure (ibid). Beyond the farm gate, biosecurity extends into broader governance structures which are implemented by both public and private sectors. Such structures include surveillance systems which actively and passively monitor disease across domestic and international domains and the legislative systems which regulate the movement (or not) of livestock within food systems. The resulting politics of biosecurity, which exist across parliamentary and policy discourse and at the science-policy interface, attempt to influence the day-to-day operational practices on farms (Donaldson 2008). Underlying all these interventions is a logic of exclusion, keeping disease ‘out’ of both physical and political spaces (Foucault 2007), a logic which has been increasingly challenged due to the complexity of livestock ecologies of production (Hinchliffe 2013).

In recent years, biosecurity in GB has been emphasised in response to a range of zoonotic and epizootic threats including, but not limited to, HPAI, bovine tuberculosis, bluetongue, African swine fever, and foot and mouth disease. These threats, though diverse in epidemiology and scale, share a common policy logic in which biosecurity is framed as a technical and behavioural fix, an operational response to the presumed externality of disease (Hinchliffe and Bingham 2008). Yet this framing has been called into question through social science literatures, where scholars have highlighted the uneven geographies of risk distribution, infrastructural politics, and social consequences of biosecurity regimes (Donaldson 2008, Enticott 2008, Hinchliffe *et al* 2013, Hinchliffe and Ward 2014, Dixon 2015, Jakobsen *et al* 2025a). From these viewpoints, disease is not merely an external threat to be excluded, but a relational product of the very systems, agro-industrial, economic, social, ecological, within which animals are raised and moved.

In this paper, we contribute to the discussion of biosecurity by applying arguments around pathogen management (Hinchliffe and Ward 2014, Dixon 2015), the politics of biosecurity (Donaldson 2008, Hinchliffe and Bingham 2008), and bio-insecurity (Wallace 2009, Hinchliffe 2013) to argue for a greater emphasis on the systems of production in which biosecurity emerges as an issue in the first place. Using the case study of GB’s poultry sector and the recent HPAI outbreaks, we argue that responses to disease require a broader perspective on poultry production systems and an understanding on how the system itself may lead to disease risk. To develop this argument, we first conduct a structural economic analysis of the three main economic poultry sectors, meat and egg production, which primarily relies on chickens, and the game bird sector which produces birds (mostly from imported stock) for sports hunting with a secondary utilisation as food.

Drawing on both industry data and insights from stakeholders, we analyse how biosecurity is practised and negotiated differently across these production regimes. In doing so, we identify two key entanglements, around poultry planning and the ‘insuring’ of poultry ecologies, both of which we argue need a long term and multi-sectoral approach to manage.

## 2 Methods

The aim of our study was to produce a structural economic analysis of GB’s poultry sectors to uncover factors which impact biosecurity and avian influenza. Going into this process we were conscious of three aspects of structure: temporal, spatial, and operational. Temporally, we examine how production has changed over time, considering the origins of ‘modern’ poultry in the 20th century, the industrialisation of practices, and using an approach described by Tak *et al* (2022), the mergers, acquisitions, and joint ventures between main companies. Spatially, we consider where production exists within GB and how this impacts poultry operations. Operationally, we consider the main stakeholders now present in production, their relationships, and relative commodity contributions. To do this we followed a production and distribution network (PDN) approach, described by Hennessey *et al* (2021), which extends ideas from value chain analysis (Kaplinsky and Morris 2001, FAO 2011, 2017, Alarcon *et al* 2021) to identify and analyse the nodes (people, firms) and relationships involved in commercial activity.

**Figure 1:**
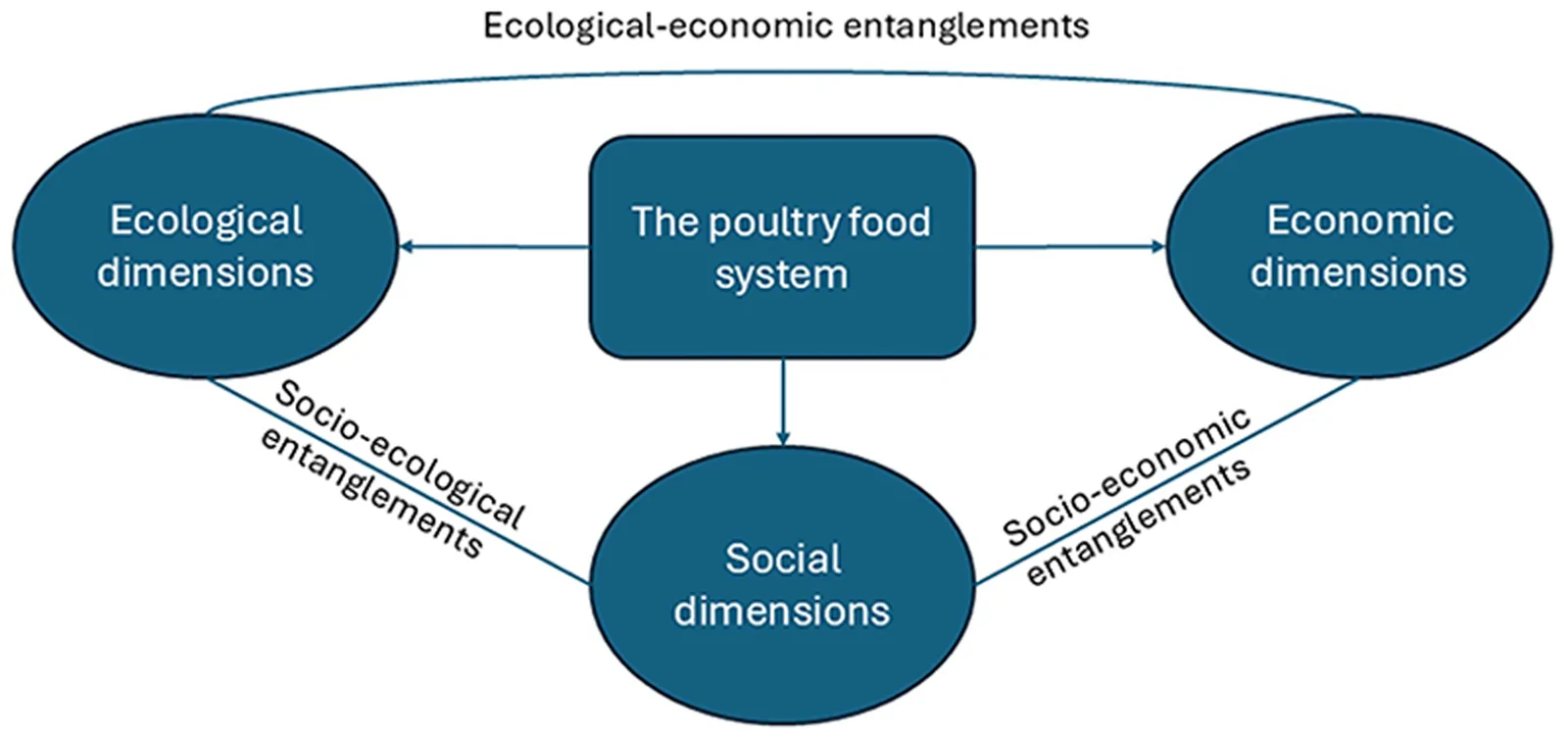
Conceptual framework for data analysis (adapted from (Francis *et al* 2003).

To identify structural factors which impact biosecurity and avian influenza, we adopted a political ecology framework (figure 1) to integrate issues concerning the environment and nature to consider the broader ‘ecologies of food production’ (Hinchliffe 2013). This approach foregrounds the socio-natural processes through which risk and inequalities can be (re)produced across people and places (Moragues-Faus and Marsden 2017). In doing so, the framework aligns with an integrative understanding of food systems as encompassing interlinked ecological, economic, and social processes operating across scales (Francis *et al* 2003).

For example, economic drivers like trade and supply chain logistics dictate the geography of production and influence local ecological habitat management. Meanwhile, social pressures, such as the demand for cheap protein and sourcing transparency, shape production economics. These intersecting forces raise critical questions about national food security and self-sufficiency, ultimately determining the specific local environments in which poultry farming occurs.

### 2.1 Data gathering and generation

The study uses multiple data sources to understand the structure of poultry production and to identify emerging entanglements between systemic factors impacting biosecurity and avian influenza (Madsen and Adriansen 2004). We collected and generated qualitative data from both reviewing relevant literature and conducting interviews, and analysed quantitative data relating to commodity production, poultry holdings, and HPAI outbreak data (the two latter sources being provided through a data sharing agreement with APHA).

An initial desk-based literature review of GB’s poultry sector was conducted using peer reviewed papers, policy documents and grey literature, publicly available information on poultry companies, and online media articles. The study focuses on GB rather than the United Kingdom^6^ due to the research focus of the wider FluTrailMap project. Data from these sources was extracted into a review of knowledge document covering the following domains: (1) production overview for broilers, layers, turkeys, ducks & geese, and game birds outlining the firms involved and production parameters (for example, the number of commodities produced, number and location of sites), (2) historical development including details of firm mergers and acquisitions, (3) details of state and private sector institutions involved in poultry production governance.

Building on stakeholder contacts created through FluMap (described in Hosseini *et al* 2025) and contacts established through the project consortium, we contacted key informants whom we expected to have high level knowledge of poultry production. At the beginning of the process, we aimed to speak with people from each of the main poultry sectors after which we followed a purposive sampling approach to contact a diverse range of stakeholders across sectors. Interviews were semi-structured, with the intention to generate information on people’s experiences of the recent HPAI outbreaks, their perceptions on the challenges which lie ahead for GB’s poultry sectors, and the factors present in the sectors impacting these issues (further details can be found in the appendix). In total, 19 in-depth interviews were conducted from November 2024 to February 2025 (table 1).

Using APHA poultry register and HPAI outbreak data (from England only), we examined the geographic densities of poultry and the proportion of each type of poultry holdings to experience an out-break of HPAI during the 2021–2025 HPAI seasons.

### 2.2 Analysis

Data extracted from the literature review was used to construct initial overview diagrams of the structure of GB poultry production and distribution. Over the period of conducting the interviews, the PDN maps were used to stimulate discussion during interviews and updated iteratively. Drafts of the maps were discussed between project members when we met on a weekly basis over the study period and shared with stakeholders to collect feedback and to improve accuracy until final versions were generated.

Recorded interviews were transcribed using an intelligent verbatim process (McMullin 2021) and entered into the qualitative analysis tool MAXQDA (ver. 24.7.0). Interview data was coded following a manifest content analysis approach (Bengtsson 2016, Kleinheksel *et al* 2020) to organise data around the conceptual framework.

Interpretation of the data we collected and generated was shaped through iterative, collective analysis within the research team, alongside ongoing engagement with the stakeholders involved in the project. Early analytical discussions focused on identifying socio-economic and ecological interactions and examining how these dynamics might influence biosecurity practices and avian influenza risk. During the initial phase of the study, several of these proposed ‘entanglements’ were further investigated in follow-up interviews to deepen our understanding of their implications. Following completion of the interviews, we continued to refine and critically assess these interpretations through internal team deliberations, as well as by sharing and discussing preliminary findings with the wider project consortium, during online industry-led stakeholder meetings, and at a final in-person stakeholder workshop^7^.

### 2.3 Ethics statement

The authors confirm that all research conducted to generate data contributing to this paper was conducted under appropriate ethics and all participants gave written informed consent to participate in the study. Ethical approval for the FluMap study was received by the RVC’s Social Science Research Ethical Review Board (SSRERB) (URN: SR2022–0130). Ethical approval for FluTrailMap was received by the RVC’s SSRERB (URN: SR2024–01000307) and the Ethics & Welfare Committee of the Department of Veterinary Medicine, University of Cambridge (ref. CR 793).

## 3 Results and discussion

### 3.1 Structural analysis of GB’s poultry sectors

Poultry production in GB spans multiple scales, species, and production systems, from small backyard flocks to highly integrated commercial operations producing meat and eggs. The sectors encompass a range of production methods, including enriched (colony) cages, barn, free-range, and organic systems (table 2) and managed outdoor environments for game birds such as pheasants, partridges, and ducks. However, certain systems dominate, which has knock-on effects for the management of biosecurity.

**Figure 2:**
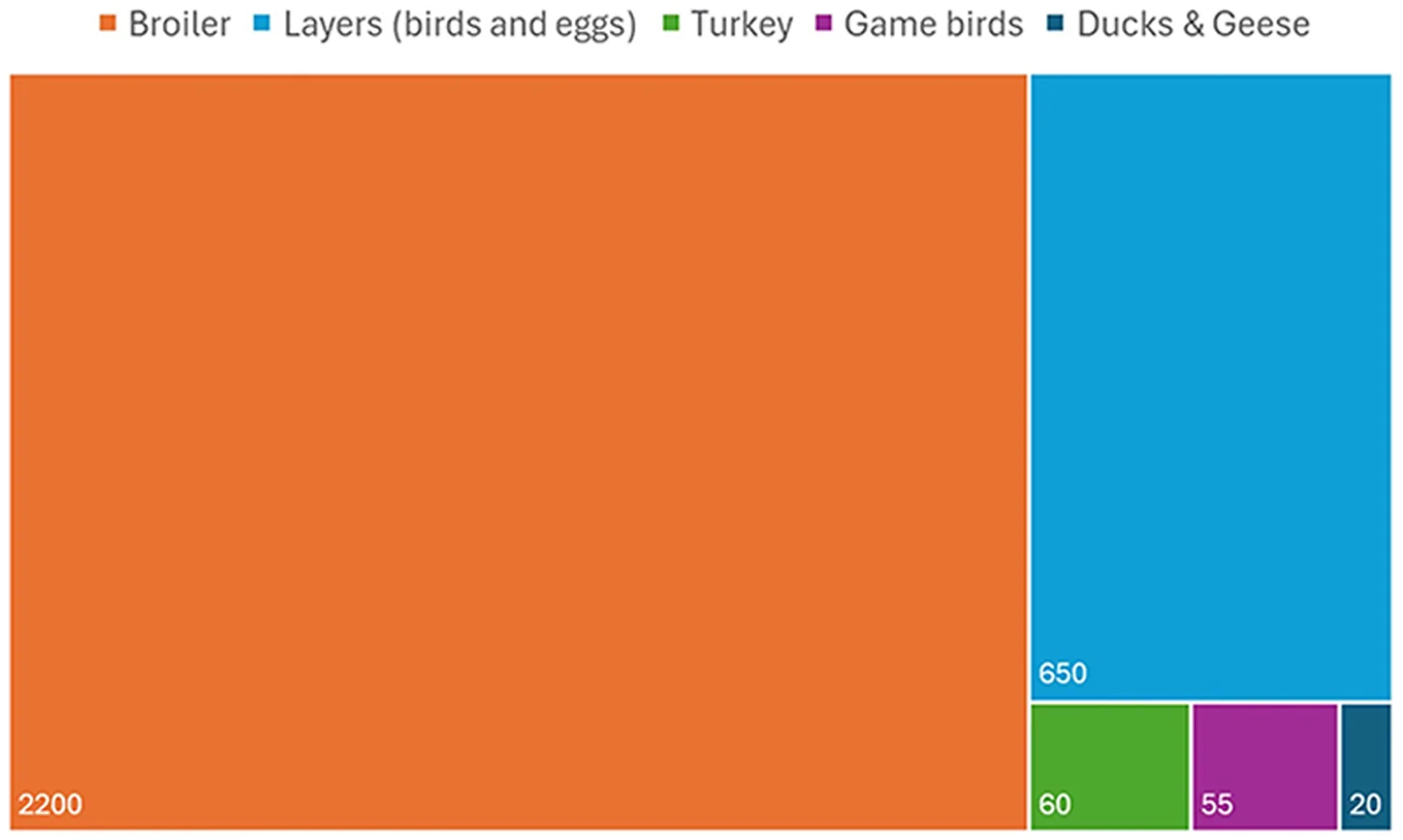
Approximate relative annual biomass production (in thousand tons) of poultry in Great Britain in 2025 (Sources BPC 2023, Red Lion 2024 and interview data).^8^

Collectively, around 1.1 billion birds are raised from eggs each year in GB to produce different commodities (BPC 2023, Red Lion 2024). Of these commodities over 90% are young chickens reared until around 35 d for meat (broilers), around 5% laying chickens (pullets) producing the 12 billion eggs for human consumption, with the remainder comprising turkeys, ducks, geese, and game birds (figure 2). While game birds are primarily raised for shooting for sport, a proportion of these birds go on to enter the food chain, supplying both domestic and export markets.

The relative economic contributions, however, reveal a different picture. Poultry meat production (chicken, turkey, waterfowl) is estimated to contribute around £5 billion annually to the domestic economy directly and from industry spending on goods and services in the supply chain (Oxford Economics 2024) and the layer sector has been described as worth £1 and 2 billion annually (BEIC 2021, Red Lion 2024). The game bird sector, while having a relatively smaller biomass contribution within overall poultry production, is thought to be worth £2–2.5 billion annually to the national economy and has been described as ‘significant rural activity throughout the winter’ (GFA, n.d.). While game birds themselves are worth modest amounts of money when sold (£4–6.50 a bird), large amounts of supplementary income are generated through the activities that surround shooting.

**Figure 3:**
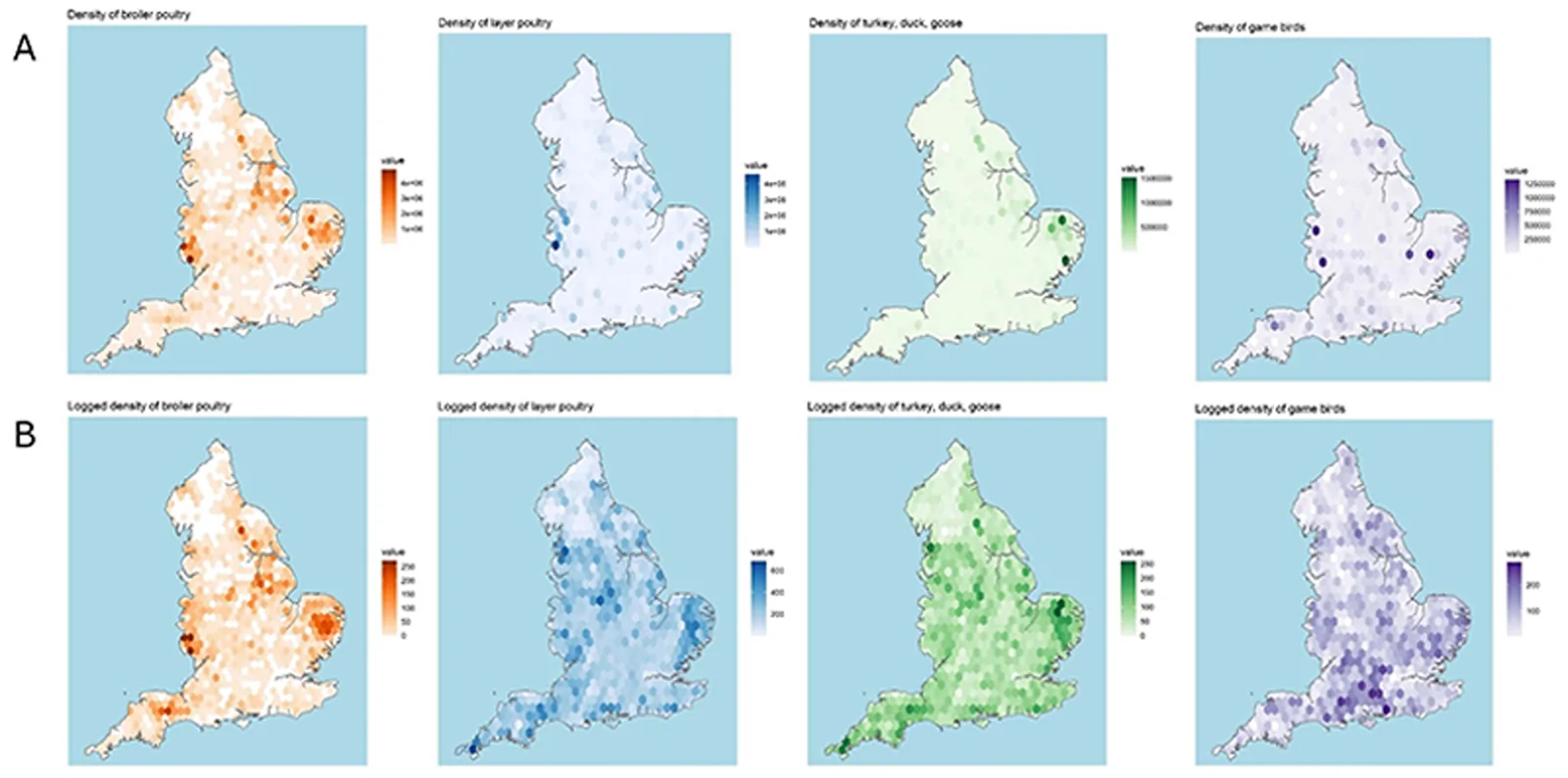
Spatial concentrations of poultry types in England. (A) Areas with peak concentrations, driven by key producers with very large numbers of birds on single holdings. (B) Distribution using log-transformed holding numbers, which reduces the dominance of extreme values and enhances the visibility of moderate and dispersed concentrations, allowing broader spatial patterns in poultry density to be identified.

Across England, where most HPAI cases have occurred over the past five years, poultry are concentrated in specific areas depending on the production system. Meat production is primarily concentrated in the west and east parts of England, particularly around the Welsh border and Lancashire in the west, and East Anglia, Yorkshire, and East riding in the east. Other notable concentrations include some large laying farms and game producers in the west of England, and concentrations of game being held in sites across the Downs National Parks and the counties of Wiltshire, Hampshire, and Sussex. (figure 3).

In the following section we describe the historical and economic development of the three largest commercial sectors, broiler, layer, and game bird production and consider what this means for poultry ecologies of production, biosecurity, and HPAI.

#### 3.1.1 Meat production: from smuggled Easter eggs to one billion broilers

The structure of GB’s broiler industry has evolved rapidly over the last 70 years, shaped by a combination of retail power, technological innovation, and market consolidation. In their analysis of the evolution of the sector, (Godley and Williams 2007) describe how post-war Britain experienced a culinary and industrial shift, with the rise of indoor reared chickens for meat marking a decisive break from earlier food systems. While the United States pioneered poultry agribusiness through the department of agriculture-led innovation and was driven by up-stream actors such as feed companies, integrators, and processors, GB’s growth was driven primarily by retailers. Companies like J. Sainsbury and MacFisheries coordinated early production changes in the 1950s and 1960s, introducing a ‘group system’ in which ‘farmers had fixed contracts with packing stations, which in turn had fixed contracts with retailers’ (Godley and Williams 2007, p 18). This framework stabilised supply and ensured greater standardisation but also established the structural power of retailers and processors in shaping sectoral growth.

**Figure 4:**
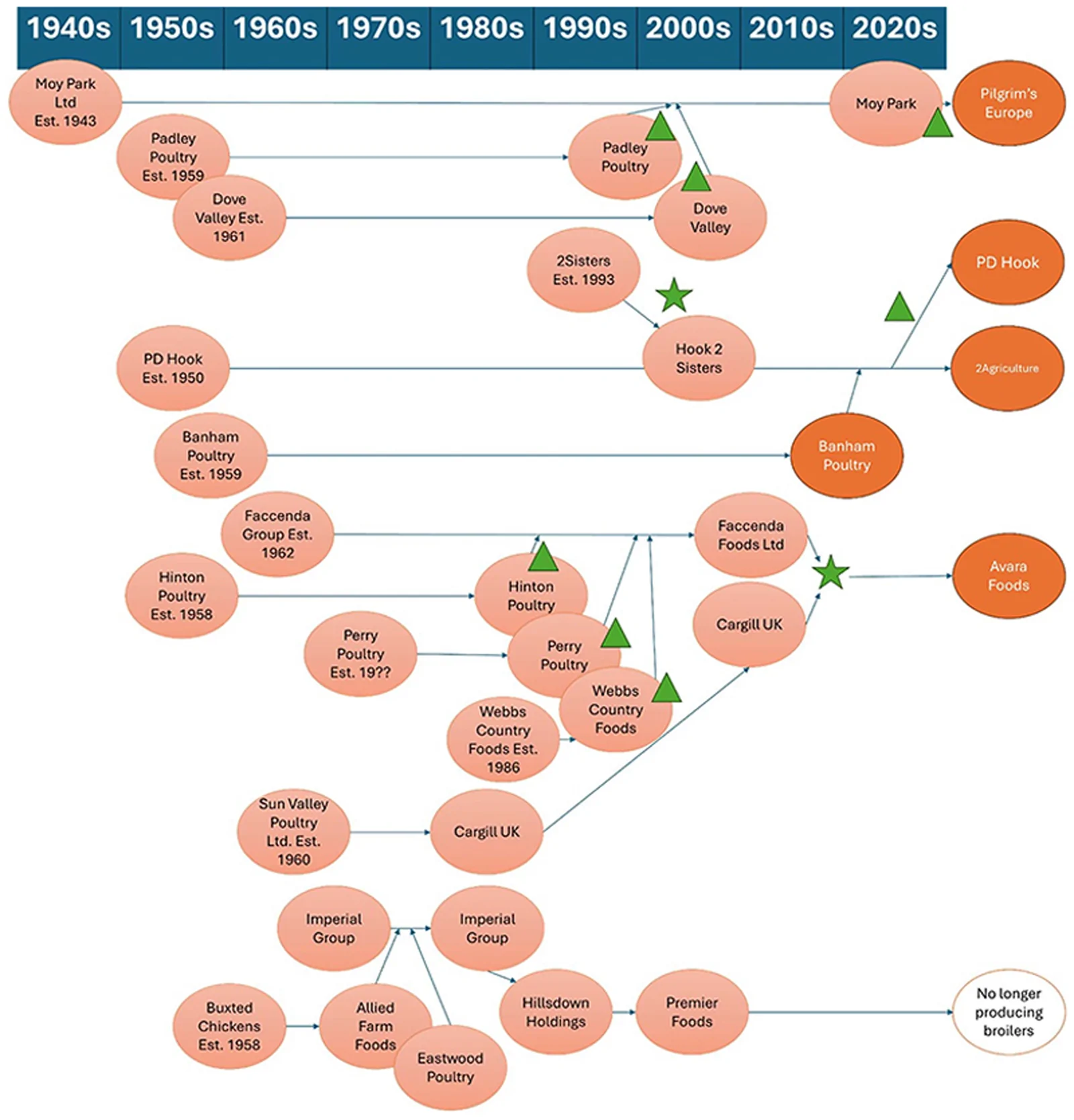
Timeline of acquisitions (green triangles), joint ventures (green star) of the main broiler companies operating in Great Britain. (Sources; information from company websites and industry reports).

One notable moment in this early history reflects the entrepreneurial energy behind GB’s modern broiler industry: Anthony fisher, founder of Buxted Chickens, smuggled fertilised eggs from the United States in 1952 disguised as Easter eggs to build GB’s domestic breeding stock (Godley and Williams 2007, p 7). Through subsequent decades, processors like Buxted expanded their reach, integrating hatcheries, feed production, and distribution into vertically aligned systems. Since then, the broiler industry has undergone repeated mergers and acquisitions (figure 4), resulting in deeper vertical integration, narrowing of control over breeding, and concentration of production and processing.

One key feature of GB’s broiler production is its genetic uniformity. Like global markets (described by Tak *et al* 2022), GB’s production relies almost entirely on the Ross 308 breed, owned by Aviagen, part of the EW Group. Ross 308 are fast growing birds, reaching a slaughter weight of around 2.2 kg in 35 d. Such rapid growth can result in higher rates of sudden death, contact dermatitis, and locomotor problems compared to slower growing breeds of broiler (Nicol *et al* 2024). Consequently, in recent times social movements have formed, such as the better chicken commitment (BCC), pushing for poultry firms to shift towards slower growing breeds, such as Redbro^9^ from Hubbard (a subsidiary of Aviagen), in an attempt to achieve higher welfare production. A small number of UK retailers and food outlets initially committed to meeting the BCC standards (BCC 2026), a move supported by the UK Government’s Animal Health & Welfare Pathway (Defra 2025a). However, eight restaurant groups representing 18 fast-food brands in the UK have recently withdrawn this commitment, citing supply chain constraints and concerns about the sustainability of producing slower-growing birds (Martin 2026).

**Figure 5:**
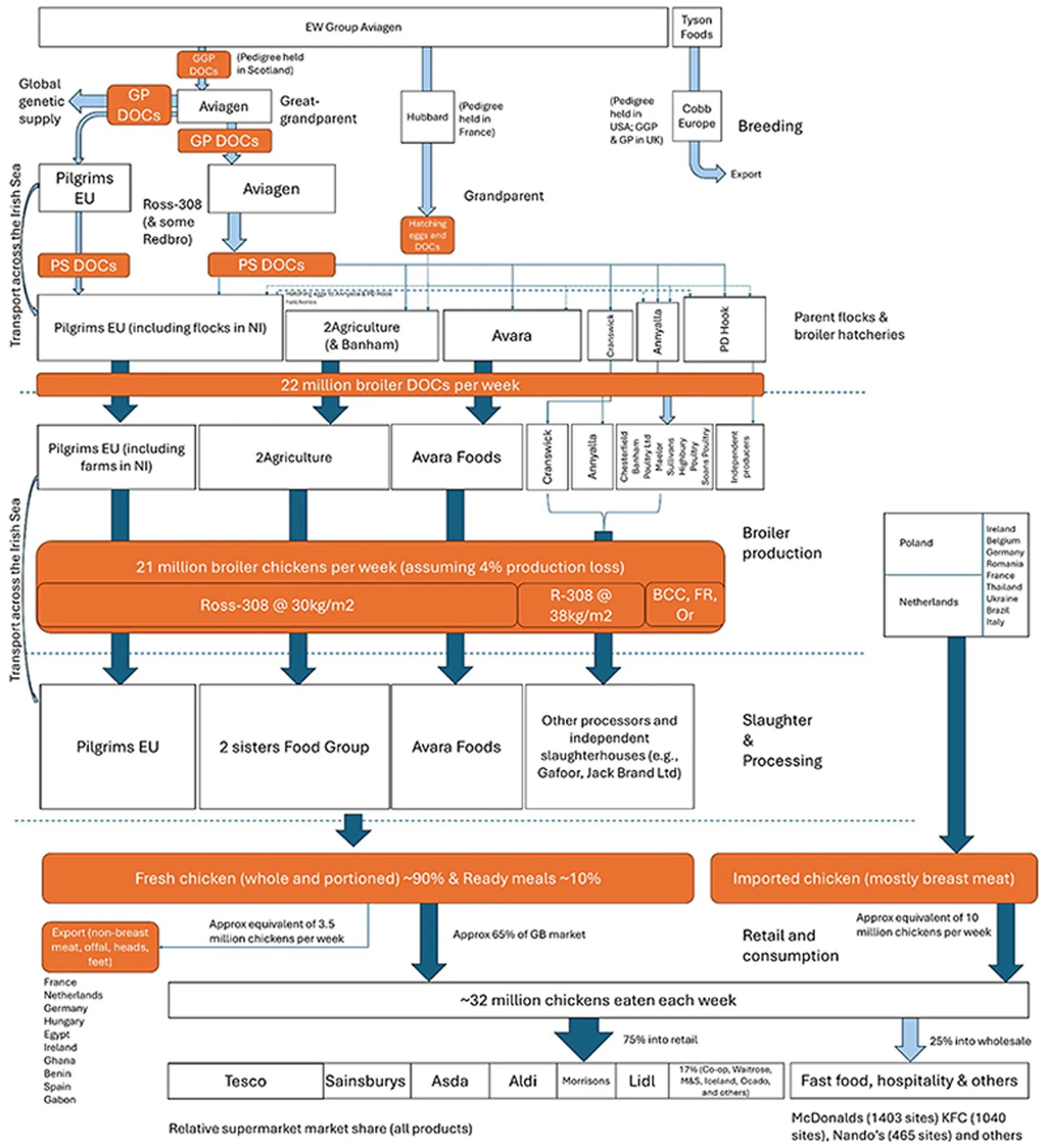
Great Britain’s broiler production and distribution network. NB. GGP—Great-grandparent, GP—Grandparent, PS—Parent stock, DOCs—Day-old chicks, NI-Northern Ireland, BCC—Better chicken commitment, FR—Free range, Or—Organic

Today, GB’s broiler production is controlled by a small number of vertically integrated companies, primarily Pilgrims Europe, PD Hook, 2Agriculture^10^, and Avara Foods, which collectively produce and rear almost 75% of the broilers produced. The remaining 25% of broilers are produced by at least 10 smaller companies (figure 5). Aviagen holds pedigree stock in Scotland which is used to produce great-grandparent and grandparent flock day-old chicks, many of which are exported to supply global genetic supply chains.

While our study focuses on poultry production in GB, it would be remiss not to mention the highly interconnected relationship with poultry in Northern Ireland (NI). Britain’s poultry industry operates through supply chains that span the Irish Sea, with breeding stock, hatching eggs, chicks, and processed meat routinely moving between regions. Major integrators, such as Pilgrims Europe^11^ which is the largest broiler firm in NI, manage production networks that link farms, feed mills, and processing facilities in both territories. A proportion of the breeding stock produced in Scotland is transported across the Irish Sea to Pilgrims Europe’s parent-stock and grower farms in NI before broilers are transported back across the sea to supply a significant share of meat to GB’s retailers and food service industry.

Poultry firms in the upstream part of the PDN oversee the supply of chicken to a similarly concentrated downstream retail sector, led by Tesco, Sainsbury’s, and Asda. Working across these firms are the farm assurance schemes, such as red tractor, which, as this poultry organisation representative noted, developed as a response to food safety concerns in the 1990s:

*‘Following the wake of BSE, where restaurants were not serving British beef because [of a] lack of trust in standards in the UK, you then had a lot of sectors setting up their own standards. [Assurance] was a way to amalgamate all those standards, which were under one logo to provide standardisation and consumer brand recognition. […] [Assurance schemes] became [their] own entity with [their] own decision-making powers, processes and governance, that’s now morphed into covering environmental concerns and animal welfare.’* ^12^

Between these dominant actors sit independent and contract farmers, who operate under conditions largely determined by processor–retailer requirements and governed for compliance by assurance schemes. As this poultry organisation representative described, assurance schemes help to manage the tensions created by what producers and retailers desire and how this can be implemented on farm:

*‘What the industry feels they could tighten up on biosecurity is a big conversation in the last couple of years. […] Where you get pushes and pulls, obviously it is really challenging because a lot of the time, retail would like everything under the sun for no additional cost. And so [assurance schemes] help facilitate those conversations and provide a commonality that allows farms to adhere to a single standard without additional audits.’* ^13^

Currently, more than 95% of GB broiler production occurs in intensive indoor systems, with only around 2.5% coming from organic or free-range operations which stipulate birds have access to the outdoors (RSPCA 2022). As this poultry firm employee described, there are socio-economic factors limiting the expansion of outdoor raised broiler systems:

*‘[Outdoor chicken] has been squeezed and gone backwards. Probably more to do with the cost-of-living crisis, I think, than [avian influenza]. There’s a lot more higher welfare indoor chicken now, like Marks and Spencer’s high welfare indoor, that puts the squeeze on the top end of the market. […] The cost of production of free-range broiler is proportionately much higher than say free range eggs [which] already have that momentum.’* ^14^

While housing birds indoors provides physical barriers between circulating HPAI in wild birds and commercial poultry, the integrity of such barriers may not always be secure. A recent survey from the National Farmers’ Union (NFU) found the average age of broiler sheds to be 31 years, with 15% of houses currently in use being at least 50 years old (NFU 2025). Such older housing may be at higher risk of infrastructural attrition, experience water ingress from leaks or flooding, creating challenges in maintaining physical and operational biosecurity.

While free-range and organic broiler production remains low, the industry has begun to shift to a lower stocking density (from 38 kg of bird per square metre down to 30 kg) for indoor birds in an attempt to improve bird welfare. The implementation of such initiatives was described by this veterinarian to be impacting production through access to a key resource, space:

*‘It is great from a welfare perspective, going from 38 kilos to 30 kilos per metre squared, but if we are going to keep the same level of UK production [and] consumption, that’s an awful lot more sheds we are going to need. And I do not think we have fully reconciled how we are going to do that.’* ^15^

If indeed a significant amount of additional space is soon to be needed, the challenge for the poultry industry is to consider where this space will come from.

Looking to the past can offer valuable lessons. An expansion of broiler farms occurred over the last decade in the Wye valley region of western England and eastern Wales, with numerous planning applications rousing public engagement and objection. Ensuing media coverage documented the controversy over concerns for how this development would impact local environments and people’s lives (Caffyn 2021). More recently, high-density broiler and layer farms within the Wye valley have been implicated in environmental degradation, including nutrient overload due to poultry manure run-off from farmland (Ungoed-Thomas 2020, Reid 2022). Currently (as of July 2026), a landmark group litigation is progressing through the High Court, brought by more than 4500 local residents and businesses against Welsh Water and Avara Foods regarding the ecological degradation of the river Wye catchment (Dearnaley 2026). The eventual findings and outcomes of this case are likely to have profound impacts on the socioeconomic-ecological entanglements surrounding GB’s intensive poultry systems. These very public concerns have galvanised local and national social movements, with campaign groups mobilising and forcing judicial reviews of council decisions to approve poultry development (Clarke 2024). In their ‘Broiler Battles’ study, Caffyn (2021) notes how local communities mobilise against poultry development on grounds ranging from odour and traffic to ecological degradation, highlighting the multi-scalar nature of resistance and the interplay of rural ideals and environmental justice. Such controversies are no longer confined to the Wye valley with similar arguments being used to resist poultry expansion in other parts of England, particularly in East Anglia (Laville 2025a).

Other poultry meat being consumed in Britain includes turkey, duck and goose. Turkey and goose production differs from broiler chicken in that birds are older when slaughtered (3–6 months compared to 5 weeks for broilers), providing a longer potential time for virus exposure. Ducks, while having a relatively young age when slaughtered (2 months), differ in that they require repeated additional straw bedding to be added throughout their growing period, increasing potential opportunity for disease incursion. Additional PDN maps for turkey and waterfowl can be found in the appendix (Figures A1 and A2).

#### 3.1.2 Egg production: an alternating relationship with the ‘outside’

Large-scale commercial egg production in GB developed after the Second World War once animal feed shortages and egg rationing ended (BEIC 2023). In the initial post-war period, around 80% of eggs were produced in traditional farmyard settings with the remaining coming from the emerging industrial sector (Harrison 1964, Mead 2023). Subsequent industrial growth and adoption of battery cage systems was such that by 1980 only 1% of UK eggs produced were free-range, meaning that, for a time, most of the UK’s hens were housed indoors. However, growing social movements around animal welfare such as those highlighted by Harrison (1964), drew attention to the severe confinement of hens in battery cages, alongside rising concerns about the safety of eating eggs in ensuing decades^16^, led to changes in both consumer demand and regulation.

From the mid-90s there occurred a re-emergence in free-range systems^17^ which now dominate national production. A change, as this veterinarian noted, partly driven by the retail sector:

*‘In going for so much free range, [the sector] has created a difficulty. […] It is been driven by the supermarket sector; it was not government policy. If you look at what government was putting into place, it was in line with the EU, enabling better housed bird production in colony cages. But the supermarkets marketed free range in such a way that, inescapably, the industry got drawn into free range.’* ^18^

**Figure 6:**
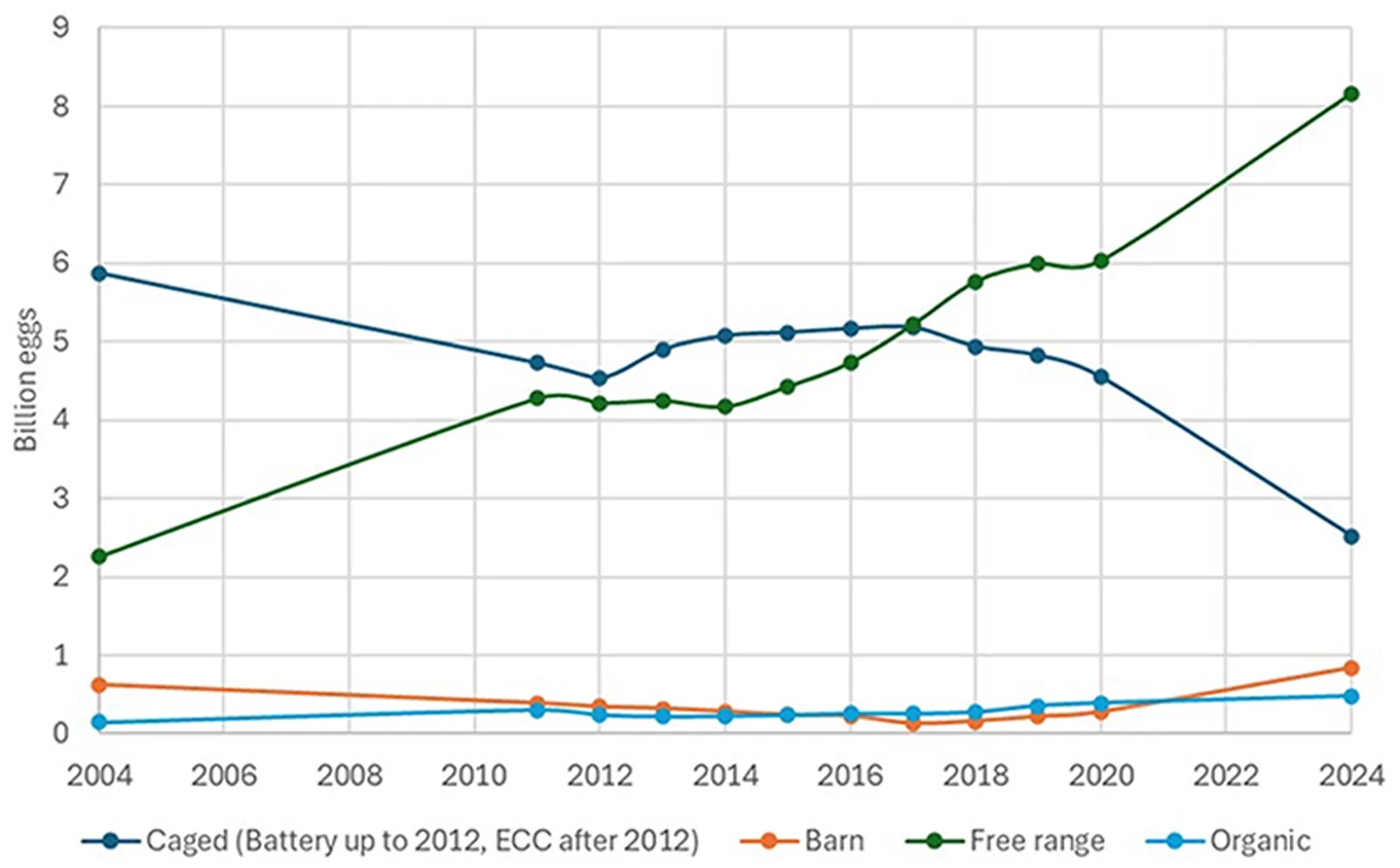
Egg production system in the UK by production system. Source (British Lion 2024, Defra 2025b).

Subsequent European Union legislation banning battery cages, which came into force in GB in 2012, brought significant structural consequences for the industry (figure 6). Initially, the implementation of enriched colony cage systems saw an uptake in cage production, however, this has been in decline over the last decade with an uptake in free-range production.

While indoor systems can house up to 13 birds per square metre, modern free-range systems require each bird to have additional access to four square metres of outdoor space. Thus, the last 70 years of egg production in GB sees a changing geography of production with varying access to ‘the outside’, interactions between farmed birds and nature, with eventual consequences for biosecurity and HPAI. As this egg producer reflected, a question remains around what the ongoing challenge of HPAI will mean for the future of egg production in GB:

*‘In my lifetime, birds have been outside. Then they went inside. Now they are going back outside. I do not know how long it will be until they are back inside again. We do not want to pay too much for food; we do not want to give up too much ground for hen houses. [With indoor production you] have more control.’* ^19^

Egg production in GB being dominated by free-range production is at odds with most of Europe, where production mainly comes from housed hens (Gautron *et al* 2022). Germany, for example, has eschewed caged systems (4.3% of total production), and most production occurs in barns (58%) with free range and organic accounting for around a third of production (Negrici 2024). Thus, GB’s laying hen flock has a relatively unique relationship with the outside compared to many neighbouring countries (table 3), with consequences for HPAI management and supply chains during times of outbreaks. During the 2022 HPAI outbreaks, national housing orders were in place for long enough that eggs from housed free-range flocks had to be re-labelled as barn eggs from the 21st March to 2nd May (Defra 2025b). However, following lobbying from the retail sector and the poultry industry, this legislation was amended in 2024 meaning that eggs from housed free-range flocks can continue to be labelled so even if they are housed for longer than 16 weeks^20^ (Defra 2024).

Most eggs in GB are consumed as ‘shell eggs’, eaten whole either at home or within the hospitality sector, while approximately 15% are utilised as liquid egg in the food manufacturing industry (British Lion 2024). Although imported eggs (predominantly from Poland and Ukraine) typically fulfil the demand for liquid egg, domestic supply disruptions can alter this dynamic. For instance, during recent HPAI outbreaks, a deficit in domestic shell eggs triggered a 60% surge in imports, driving foreign shell eggs onto GB shelves and tables (BEIC 2026). This heightened reliance on imports has sparked concerns regarding food safety and animal welfare, particularly if these products originate from systems lacking rigorous *Salmonella* controls or utilising conventional battery cages banned in GB.

**Figure 7:**
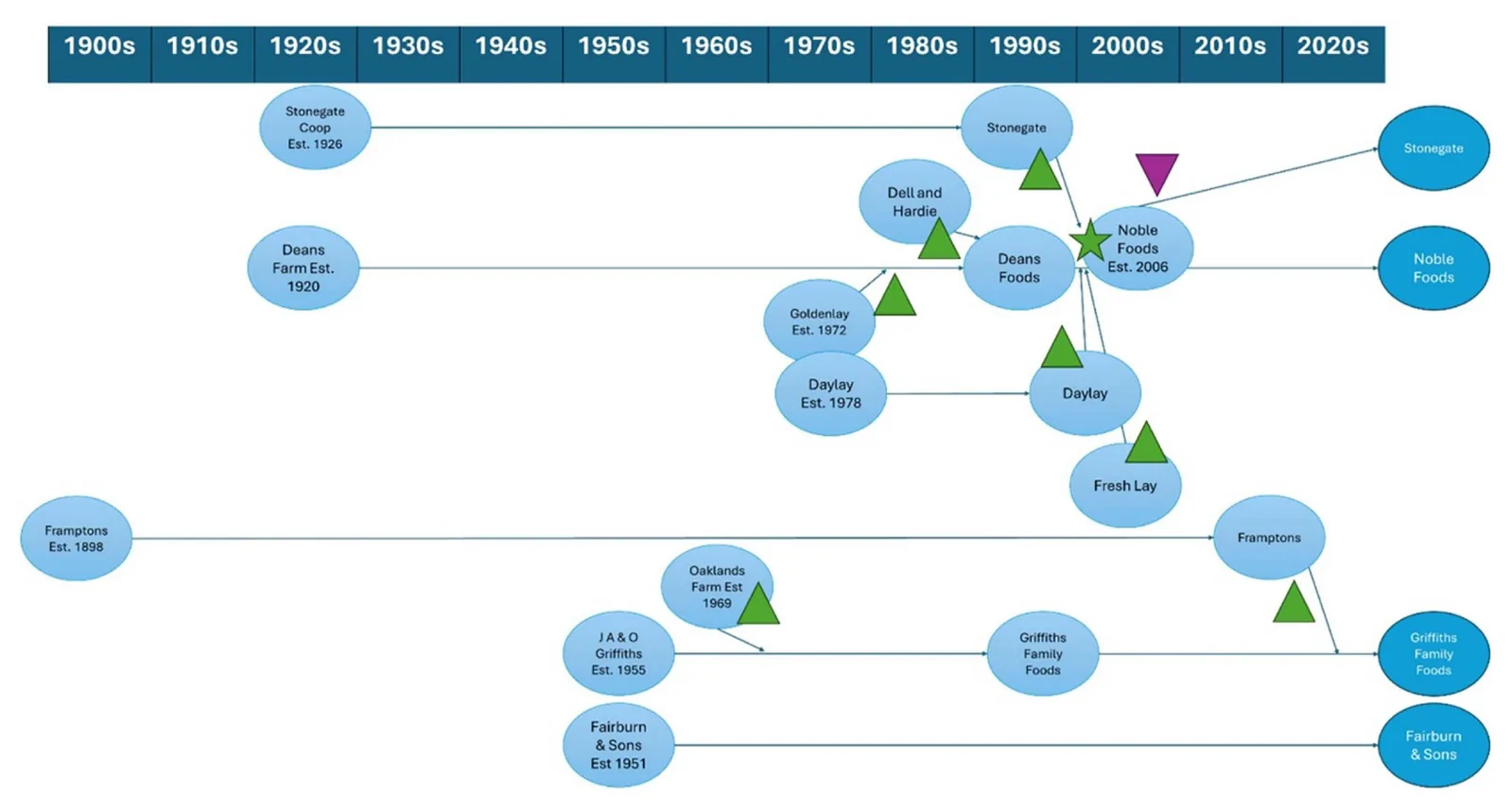
Timeline of acquisitions (green triangle), mergers and rebranding (green star) and divestments (purple triangle) of the main four layer companies operating in Great Britain (Sources; information from company websites and industry reports).

Like the broiler sector, most production of egg laying chickens in GB originates from a small number of companies. Country Fresh Pullets, Noble foods, Stonegate, and Potters Poultry supply 16 week-old birds (pullets) to laying farms around the country. Numerous mergers and acquisitions have occurred over the last 40 years resulting in this concentration of production (figure 7). Of note here is how the UK Competition Commission forced the divestment of Stonegate by Noble foods in 2007 due to concerns that this acquisition would allow Noble foods to hold too dominant a market position, with potential negative consequences for retailers and consumers (Competition Commission 2007). While over half of GB’s eggs are processed through 35 companies, the four largest firms control around 48% of eggs produced indicating a moderately concentrated supply chain, albeit less so than the broiler sector.

A key relationship in egg production is between producers and egg packing centres. As this insurance provider described, this relationship has shaped economic activity and poultry geographies, situating production near processing, resulting in potential supply chain vulnerability should avian influenza occur:

*‘Processing plants want [producers] nearby because they have got rules about how long someone can be in a lorry, and they cannot be away in the middle of nowhere. If you take Noble food, the biggest egg packer that we have got in the country. They have got loads of [layer] sheds around their packing facilities in Lincolnshire and from their side, back in the day, that obviously made perfect sense because the chickens are right there. It is only half a mile up the road. [But if] suddenly [the farms] come down with avian influenza, the factory’s got [movement restrictions] right around that says you cannot bring anything in or out.’* ^21^

**Figure 8:**
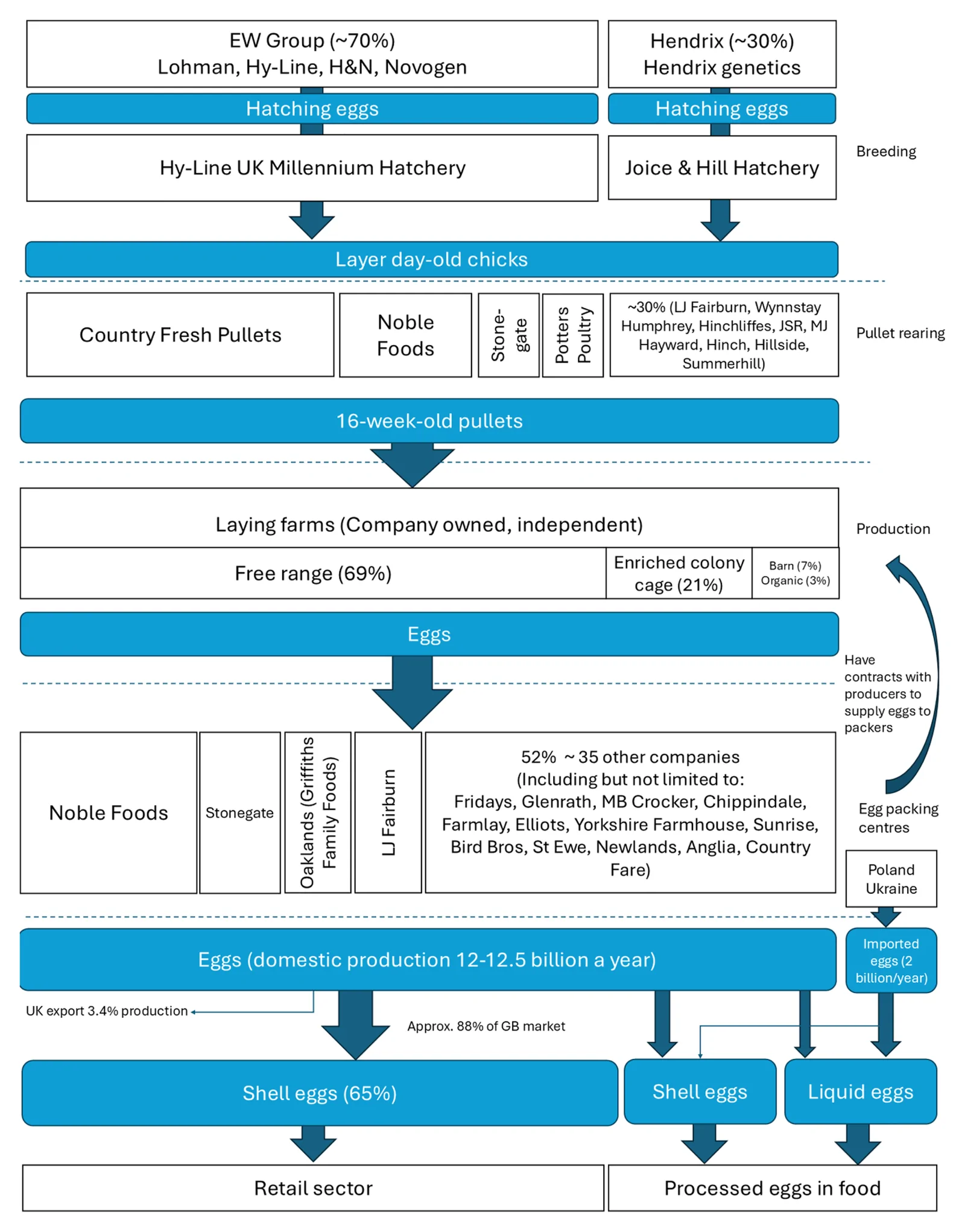
Great Britain’s layer production and distribution network.

The two main domestic hatcheries, Hy-Line UK (supplied with hatching eggs from the EW Group) and Joice & Hill (supplied by Hendrix Genetics), provide the day-old chicks required to maintain GB’s national flock of 43 million birds (figure 8). Consequently, the market concentration of layer genetics by just two dominant companies mirrors both the global landscape described by Tak *et al* (2022) and the structure of GB’s broiler sector.

Since 2016, numerous parts of the GB’s egg sector have signed up to the voluntary ‘Cage-Free 2025’ commitment which seeks to cease the use of enriched colony cages altogether. Noble Foods, for example, the UK’s biggest egg producer had plans to replace its enriched colony cages with aviary barn systems (Noble Foods 2020a). Though, in an online video describing their approach to cage-free 2025, they note the expense of such a move:

*‘Free range is not necessarily the cheapest way of producing eggs. So, when we look at that we have to consider and balance welfare against the cost of production. One of the challenges for us as a business is we invested heavily in the enriched colony system that was previously in this house. So that was brand new in 2009. But this is about creating a system which we feel is fit for purpose going forward into the future.’*(Noble Foods 2020b)

Similarly, others, such as this poultry industry representative, shared concern over the cost of cage-free 2025 and who should pay for it, and as with the move to a reduced stocking density in broilers, noted the space considerations involved:

*‘The enriched units tend to be big hangar-like buildings, but with no land [available], you cannot go free range. So, you have to go to barn. And you know, we spent $400 million [as an industry] moving in 2012 to get rid of battery cages in this country. All industry money.’* ^22^

Currently the move to cage-free production is being driven by the major retailers, who have responded to social movements and consumer demand. Consequently, this initiative remains primarily focused on the production of shell eggs destined for retail. Thus, without an upward market pressure to change, it is likely that those supplying eggs for the food industry may not yet adopt this potentially costly change. The Department for Environment, Food & Rural Affairs (Defra), however, recently launched a consultation on outlawing all cages for egg production which would then impact all producers across GB (Defra 2026b).

#### 3.1.3 Game birds: commercially produced wild birds

GB’s game bird industry occupies a distinctive position within the national poultry landscape, sitting at the intersection of agriculture, leisure, and wildlife management. Unlike broiler or layer production, which are structurally embedded within consolidated food systems, game bird enterprises service a recreational hunting economy centred on pheasant and red-legged partridge shoots, with smaller contributions from mallard ducks (Madden 2020, Aebischer 2024). Commercially reared game birds now underpin most organised shoots in England and Wales, reflecting a long-running industrialisation of what was once a predominantly estate-based activity. Tapper (1992) notes how land reformation occurring in 17th century Britain, brought about by the enclosure acts, created an ideal landscape for game. By 1850, much of rural Britain had become enclosed land, separated by hedges which provided shelter and nesting ground for pheasants and partridges, with rotation arable crops being farmed which created habitats for birds to forage. Upon this landscape, access to hunting was governed through the Game Act of 1671 (repealed in 1831), which restricted rights to hunt to the wealthy elite, effectively criminalising poaching and making game a symbol of aristocratic power (ibid).

**Figure 9:**
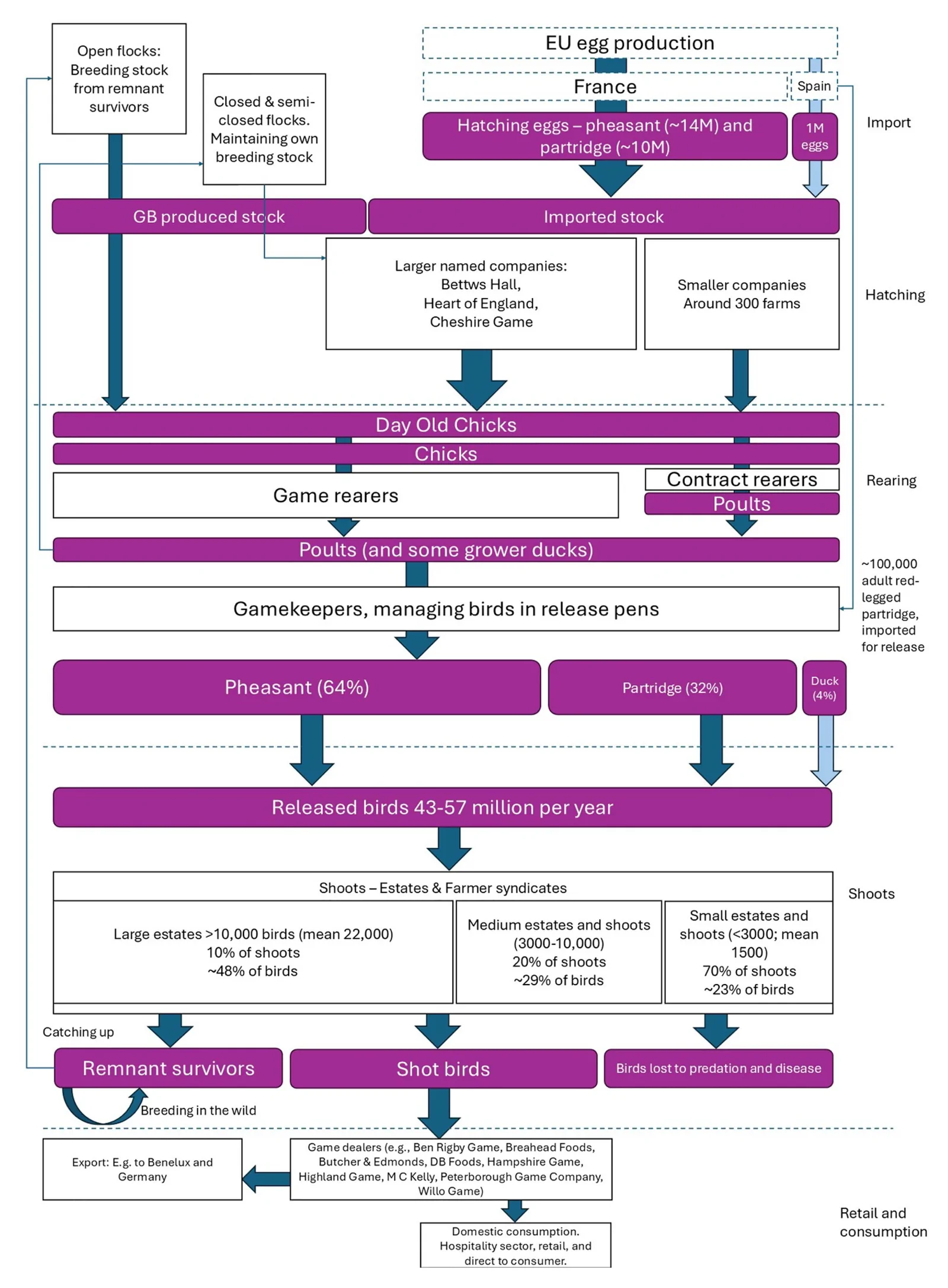
Great Britain’s game bird production and distribution network (numbers are in birds per year).

Modern game production systems involve the hatching, rearing, and release of an estimated 43–57 million birds each year across rural landscapes (Madden 2020, Mason *et al* 2020), supported by specialist hatcheries, feed suppliers, and contract rearers (figure 9). Today, shooting game is governed by the Game Act 1831 and the Wildlife & Countryside Act 1981, and requires landowner permission, shotgun licences, and can only occur within certain periods (table 4).

Most game birds produced in GB each year rely on imported hatching eggs, primarily from France (with a small proportion coming from Spain, Poland, and the USA). During 2022, HPAI outbreaks in Europe meant that under UK law it was not possible for these imports to reach GB, resulting in negative economic impacts on the sector (Countryside Alliance 2022). Consequently, some domestic companies looked to change their production models, relying more on their own breeding stock, either by maintaining closed flocks, or by using remnant survivors for breeding. Implementing such change, however, after two years of economic disruption due to COVID-19 restrictions, proved challenging for producers in the sector, and reliance on imported stock still dominates.

Young game birds are reared within controlled facilities, often under husbandry conditions not dissimilar to small-scale broiler and layer systems. Release occurs during late summer prior to the start of the shooting season, into semi-wild release pens where interactions with wild bird populations become unavoidable. Once birds move out of the release pens they are classified as wild and, as this veterinarian noted, are no longer under the jurisdiction afforded to commercial poultry, which creates challenges for biosecurity management:

*‘It is a huge sector and these birds; I guess they sit in a weird box because they are not quite wild. They are not quite domestic. […]. I mean, the good thing is that most of the game birds are reared between the start of April and the end of August when we have got the least amount of avian influenza floating around.’* ^23^

Since 2021, releasing game birds on or near to special areas of conservation requires producers to apply for a general licence 43 (GL43) and for those within 500 m of a special protection area to apply for an additional licence, the GL45. In early 2025, however, Defra elected not to issue the GL45 licence for the upcoming season, due to ongoing concerns regarding HPAI, instead requiring producers to apply for individual release licences from Natural England (BASC 2025a). While the British Association for Shooting and Conservation (BASC) took legal action to force a judicial review on the matter (BASC 2025b), Defra’s decision to halt the GL45 licence did not change.

Game birds cannot be released from sites under HPAI protection or surveillance zones or when national AIPZs with mandatory housing orders are in force (Defra 2025c). Such national housing orders, however, do not normally coincide with the late summer game release period and so, to date, the annual cycle of game bird production and release has largely remained unaffected by HPAI. Should GB experience summer HPAI outbreaks on the scale of, or exceeding, those seen in 2022, any such national housing order could have devastating consequences for the game bird sector.

Defra’s guidance on avian influenza viruses (AIV) and game birds contains recommendations around feeding including placing ‘feeders in areas that are less attractive to wild birds’ and removing ‘spilled feed daily to avoid attracting wild birds and wildlife’ (Defra 2025c). These recommendations, which are arguably vague and difficult to enforce, highlight how the boundary between agriculture and nature is especially challenging for game bird production and HPAI, where risk pathways differ markedly from enclosed broiler or indoor layer farms.

After being shot, a proportion of game birds enter the human food chain. Birds can be taken home by people on a shoot for consumption or are taken by traders to supply domestic and international markets. Domestically, game is used within the hospitality sector (pubs and restaurants) and sold through specialist retailers. In Europe, Germany and Benelux^24^ remain key export markets for game birds.

The recent AIV outbreaks have highlighted tensions between the state and the game bird sector and generate consideration over how this primarily recreational sector fits into GB’s broader poultry ecology. In addition to the game sector’s contribution to rural and national economies, the role of game estates in conservation is contested. Some studies highlight the positive ecological impact of game management on habitat quality and in countering biodiversity loss associated with modern farming techniques (Mustin *et al* 2018, Mason *et al* 2020). Others, however, highlight the negative ecological impacts of game birds. Estimates suggest that tens of millions of game birds are released annually in GB, rivalling the biomass of native wild bird populations during peak periods (Blackburn and Gaston 2018). This scale of release indicates that pheasants, which account for almost three quarters of released game birds, play a significant structural role in local ecosystems (Blackburn and Gaston 2018, Madden 2020). Furthermore, pheasants have been found to be particularly susceptible to certain HPAI viruses and to act as efficient transmitters of infection to chickens (Seekings *et al* 2024). Questions therefore emerge over how this historically private, leisure-oriented sector should be positioned within national disease resilience strategies, and how future governance might reconcile ecological uncertainty with cultural, economic, and rural identity claims tied to game management and shooting.

### 3.2 Negotiating poultry biosecurity tensions

Here, we build on our structural analysis to consider how decisions concerning poultry planning and avian influenza management are the result of negotiated entanglements of social, economic, and ecological tensions and their potential impact on biosecurity.

#### 3.2.1 Planning considerations for a secure poultry ecology

The siting of poultry production in GB is shaped by a complex interplay of land access, connections with PDN, proximity to people, and environmental concerns. Each of these dimensions influences not only production viability but also broader questions around public health and social lives, animal welfare, and environmental sustainability. Currently, the poultry industry faces mounting pressures, including shifting production standards^25^, disease risks due to HPAI, infrastructural attrition, and societal resistance to expansion, which are all creating tensions around the space needed to develop new poultry sites.

**Figure 10:**
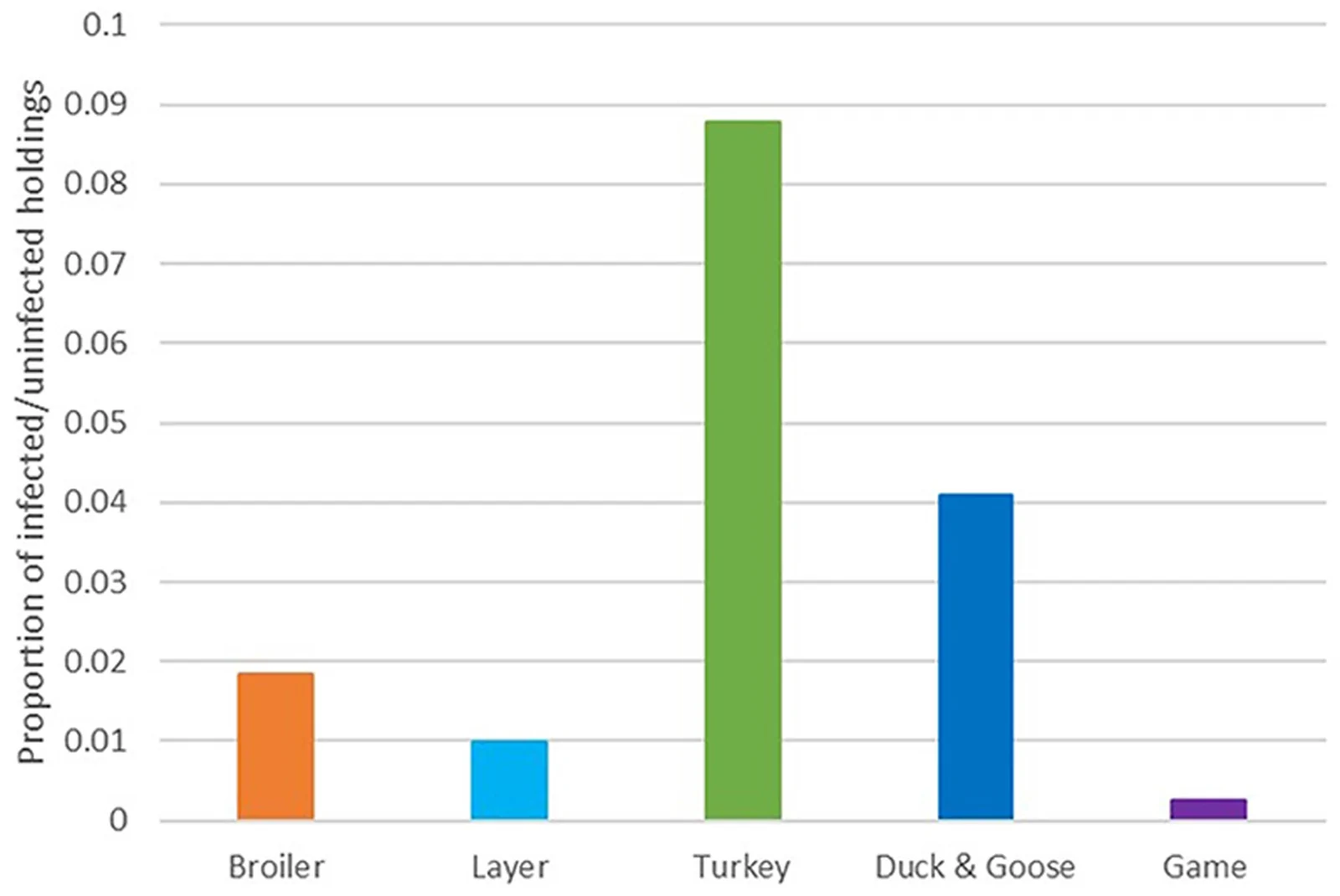
HPAI outbreak proportions during the 2022 and 23 season.

Overlaying these practical concerns of where to site poultry are epidemiological and ecological factors. Examination of APHA’s H5N1 outbreak reports reveals a seasonal concentration of cases in October to December, associated with the influx of migratory birds from continental Europe to GB, largely affecting eastern regions of England (APHA 2022). Analysis of outbreak data from 2021 to 2025 in England shows that turkeys, geese, and ducks contributed disproportionately to HPAI cases, with a higher proportion of infected premises relative to the total number of premises in those sectors compared with broiler, layer, and game bird operations (figure 10).

While there may be a range of reasons for these trends, one particular concern is the proximity of poultry-dense areas in East Anglia and the North–East of England, where turkey production is concentrated, to migratory bird pathways (Hill *et al* 2019). Here, ecological interactions between wild birds and commercial poultry need to be considered when undertaking spatial considerations of where poultry are to be sited and local governance of poultry related activities.

The processes around planning and approvals for new sites has become increasingly challenging for many in the poultry sector. As this poultry veterinarian highlighted, it is not just objections to new sites which create challenges, but also additional requirements for biodiversity investment, which can potentially impact how wild birds interact with poultry sites:

*‘One of the most frustrating things is if you want to build a chicken shed in the UK, the planners will require that you put a pond for wildlife within a couple of metres of your sheds. But from an AI point of view, it is crazy. And you know a point that industry and the [British Veterinary Poultry Association] BVPA have raised repeatedly with government is “what are you doing?”. One branch of government is saying you do not want any water near chicken sheds. But then you have got local authorities and planners saying, oh, we want to encourage the newts and everything else, which is all, you know, very admirable, but maybe do not put it right beside the chicken sheds.’* ^26^

Part of GB’s sustainable farming incentive scheme concerns water management, encouraging farmers to establish up to three ponds per hectare to ‘increase biodiversity, help improve water quality, and provide habitat for native aquatic species’ (Defra and Rural Payments Agency, 2024). Although official guidance advises against feeding waterfowl and suggests restricting livestock access to pond edges, it lacks specific directives regarding the placement of ponds near poultry infrastructure. Arguably, while these environmental incentives may bolster local biodiversity, they risk altering the movement patterns of wild birds and pathogens, and thus impact HPAI risk for poultry. The UK Government’s decision not to issue the general licence 45 for the release of game birds near special protection areas in 2025 highlights how the state is willing to intervene with a private sector to protect wild bird ecologies against HPAI. Yet, while the disease risks associated with game birds remain a key area for future research, a parallel approach must be applied to landscape management. Ultimately, without stricter regulatory oversight regarding where subsidised ponds are dug, well-intentioned biodiversity incentives may directly undermine poultry biosecurity.

These challenges reveal a deeper conflict between environmental governance, rural development, and national food security. Currently, planning applications for new poultry sites are dealt with by local authorities^27^, with national institutions, such as the High Court, only being involved to resolve appeals and tensions to planning decisions^28^. As Vergne *et al* (2024) argue in their work on biosecurity and HPAI in France’s poultry sector, current strategies of having farm level biosecurity are no longer sufficient strategies to mitigate the risk of disease emergence. Instead, the authors suggest policy makers adopt a ‘regional biosecurity’ approach which considers structural reform and the spatial distribution of poultry infrastructure. Similarly, GB’s poultry ecology requires a long-term, and national, approach to planning. Current site selection and planning processes are fragmented, often reactive, and increasingly contentious. Given the tensions of production imperatives, biosecurity challenges, public opposition, and ecological constraints, we argue that the current ad hoc, reactive approach to poultry planning is no longer sufficient. We do not advocate for the relaxation of regulatory standards, nor the sidelining of environmental and welfare concerns, but suggest and question whether a more strategic, long-term approach to planning is needed. Without such, the constraints we have documented could lead to reductions in national poultry production, which in the face of stable consumption patterns, could result in an increased reliance on imported poultry, with potentially negative consequences for national food security and commitments to animal welfare. Furthermore, these planning bottlenecks expose a critical friction between scales of governance. While national climate strategy relies on shifting consumer diets toward lower-carbon proteins like poultry as a substitute for higher-carbon red meats, local planning architectures and social movements are not conducive to this shift. Consequently, localised resistance to poultry infrastructure is likely to impede the broader structural capacity to achieve a less carbon-intensive food system (unless significant reductions in overall meat consumption occur, a point we return to later).

#### 3.2.2 ‘Insuring’ a future for poultry production

The recent waves of HPAI outbreaks in GB have exposed structural tensions within the governance of animal disease management, particularly regarding the frameworks for compensating producers for disease outbreaks, and the risk management strategies producers and farmers adopt to manage HPAI. Before 2021, several insurance firms offered HPAI coverage to producers, but as outbreaks became more frequent and severe, many withdrew from the market. At the time of our interviews, only a small number of providers remain active, and AIV policies have become prohibitively expensive for producers. For small and medium-sized operations, as this broiler firm employee noted, obtaining insurance is increasingly difficult:

*‘[It is] very difficult with [avian influenza] AI. I do not know anyone who would have AI covered in their insurance to be fair. Yeah, it is very, very difficult to meet the insurance [requirements]. I fully understand when you have a country with over 200 cases year on year, this year is not good, but, like, we do not have any insurance.’* ^29^

Some producers have tried to access insurance policies through group initiatives. One group of broiler farmers reported how they were able to do this successfully, while another free-range egg supplier described their failed attempts at this:

*‘We cannot get [AI] insurance. No, we have never had insurance, and we cannot get it. […] The insurance, actually, the underwriter has pulled out in the UK. […] There’s no point in wasting your time filling in the forms, because [they] will not entertain it. However, we do have money in the bank, and you could say we are semi self-insuring*.^30^

Larger poultry firms, like those which dominate broiler production, can self-insure through internal compensation systems, allowing them to support contracting farmers through outbreaks. While this provides a buffer, it does not eliminate all financial losses for farmers as producers lose performance bonuses and other incentives, putting them considerably out of pocket.

Differences in production cycles and environments mean that insuring game against HPAI has not been possible. Unlike broiler and egg production, where poultry housing can be restocked after outbreaks once appropriate cleaning and disinfection has occurred, similar strategies cannot be followed for the woodland environments in which game live. After the culling of game birds, the only strategy available to producers is to leave a site empty for 12 months, thus severely disrupting their annual production cycle. Consequently, outbreaks of HPAI in game birds can result in disruption which spans multiple years with severe economic consequences.

Interestingly, for those companies still offering HPAI insurance, the underwriting process itself has become a form of biosecurity governance. As this insurance provider described, assessments begin remotely, such as analysing images from Google Earth to evaluate poultry buildings, their surrounding environments, and proximity to water bodies, before moving to in-person visits or the acquisition of up-to-date photographs:

*‘I think the risks present better now than they ever did, the tools to help you sit at your desk and do it are more readily available. Ten years ago, Google Maps was not what it is now. So, someone would send us across detail about their site. I can now go [online] and have a look and talk to them and ask them about various points.’* ^31^

We suggest that a functioning insurance market for AIV could influence spatial and operational decisions concerning biosecurity. If premiums were lower for farms with a reduced relative risk of HPAI incursion, such as those located away from water bodies or migratory bird pathways, insurance markets may influence the geography of poultry production. Research on poultry insurance markets in Iran by Hasan-Nejad *et al* (2025) points to the stabilising effect insurance can have on broiler producers’ income and suggest that policymakers and industry stakeholders should prioritise strengthening insurance schemes and encouraging greater uptake among producers.

At the heart of these tensions lie the politics of biosecurity: the governance mechanisms by which early disease reporting is incentivised, and producers renumerated for animal losses. Producers and veterinarians we spoke with expressed frustration at how HPAI compensation was initially provided by the APHA. According to standard practice (as set out in the animal health act 1982), at the start of the 2021 outbreaks, compensation was only paid for alive birds at the time of culling^32^. This policy failed to account for the time lag that could occur between disease reporting, laboratory confirmation, and commencement of culling. In fast-moving outbreaks, especially in highly susceptible species such as turkeys, this could mean tens of thousands of birds had already died between an outbreak being reported and the time culling started which, as this veterinarian described, dramatically reduces the value of compensation:

*‘So how it worked to start was you’d call it in as a private vet, then the APHA would send their vet out to diagnose. They would then say yes, I agree with your suspicion and take samples. […] Then you’d get your positive result back and then [APHA would] send another vet out to do the compensation assessment. And at that point they’d then decide how many birds are left alive and how much [they are] gonna get paid. But the reality is that was often about five days down the road after the initial call, and within five days the entire lot can be dead. So, at that point, the farmer was getting compensated zero. But at the time that it had actually been called in as a diagnosis, they were maybe 87% alive.’* ^33^

However, as the outbreaks continued, the government changed its compensation procedure, to account for possible delays between a laboratory confirmation of HPAI and the time of culling. Now an APHA veterinarian must assess the health status of flocks, and the number of birds to be compensated for at the time of culling or within 48 h of the decision to cull, whichever is sooner (Defra 2022b). Crucially, the compensation scheme at the start of the recent HPAI outbreaks stood in contrast to protocols for other larger livestock, where payments are calculated based on herd size and estimated value at the time of reporting, not at the point of death or cull. This inconsistency has been a source of contention among poultry producers, leading to legal challenges. In 2024 the National Farmers Union (NFU) initiated legal proceedings against the APHA to challenge the timing of compensation, arguing that calculations should have been made on the numbers of live birds at the time a decision to cull was made and not when culling took place (NFU 2024a). While the NFU initially won the case at the High Court, the APHA appealed this decision which was then overturned at the Court of Appeals (NFU 2024b), meaning that many farmers would not get a recalculation of their previous compensation. This legal challenge reflects broader questions about accountability, fairness, and the recognition of poultry as economically and socially valuable animals, in line with other livestock.

In response to these shortcomings, several industry-led proposals have emerged. One suggested reform is the implementation of a levy-based compensation system, similar to amendments to the pig industry levy act (1983) which were introduced to support compensation in the pork sector after the 2001 foot-and-mouth outbreak (Cranborne Viscount 2001). Under this Act, pig producers would collectively contribute to a fund (in addition to contributions by state^34^) that could be used to provide more timely and equitable compensation during disease events. However, given the extent of the recent HPAI outbreaks, it is doubtful whether such funds would be sufficient to sustain an effective levy-based compensation system.

An alternative proposal, such as was described by this veterinarian, could be a tiered compensation scheme, where producers with higher demonstrable levels of biosecurity would receive more generous compensation from the state:

*‘There was discussion a while back as to whether when it comes to compensation, you actually get the high level of compensation if you have been audited and demonstrated to have a high level of biosecurity because then you have [had to] jump through all reasonable hoops to be at that level. But if you have got low levels of biosecurity, well, you did not heed what government was saying, and on your head be it. Why should government pick up the tab?’* ^35^

While such a framework could incentivise better on-farm practices, it would require independent verification and ongoing regulatory oversight, raising the question of which type of stakeholder (state, private, or hybrid) would be tasked with enforcing such standards.

These debates tie directly into broader politics over the distribution of responsibility in managing zoonotic risk and who should bear the burden of preparedness and response. Given that HPAI is a notifiable disease with zoonotic and pandemic potential, the state arguably has a responsibility to be involved to protect public health. However, as Rutt *et al* (2025) argue on their work based in Denmark, AIV compensation, along with monitoring and research activities, constitute another agricultural subsidy by the state and therefore should be a topic of public concern and require scrutiny. Gilbert *et al* (2017) and Wallace (2009) have argued that intensive production systems, characterised by high stocking densities, rapid bird turnover, and globally standardised genetics, create the very conditions in which novel pathogens like HPAI emerge and evolve. In this sense, the ‘outside’ threats that biosecurity aims to exclude are often co-produced with the ‘inside’ of industrial poultry landscapes themselves (Hinchliffe and Ward 2014, Jakobsen *et al* 2025b).

Another possibility for GB’s poultry sector would be to consider whether alternative models of production which move away from concentrated industrial production have viability. Drawing on research into Danish poultry production, Rutt and Møller (2025) argue that in the current ‘pandemic era’ of HPAI, the ‘repair’ mechanisms used to stabilise systems at risk of disease incursion do little to address the deeper structural sources of instability. The authors note that interventions largely preserve the status quo of intensive poultry farming, while sidelining smaller-scale or organic systems of production. Instead, Rutt and Møller call for a shift towards farming practices that embrace ‘care beyond repair’ which prioritise alternative models of production such as de-intensified production systems and the revitalisation of localised, slower-growing heritage breeds.

In summary, our findings call for a re-evaluation of the consequences of the state–market–producer relationship as it currently functions in managing industrial poultry, HPAI, and biosecurity. The current challenges, namely poultry planning bottlenecks, a lack of poultry-sensitive biodiversity incentives, market failures in AIV insurance, and inconsistent livestock compensation, require a joined up, multi-sectoral approach. As Hinchliffe and Bingham (2008) note, biosecurity is not simply a technical fix to an isolated problem, but an ongoing set of practices required to manage relationships between microbes, animals, and humans that co-exist in a broad ecology of production.

Such considerations could be integrated into a broader policy framework, perhaps as part of a national poultry strategy that recognises disease as a collective structural challenge rather than an individual burden to be managed on farm. Indeed, Defra, through its recent ‘Farming Roadmap 2050: Growing England’s Future’ has commissioned the development of a targeted poultry sector growth plan (Defra 2026c). Crucially, any such national strategy must deliberately navigate the complex friction of scale and governance architecture. As our data shows, a major vulnerability emerges when national-level biosecurity or environmental directives fail to accommodate regional specificities. Instead of a one-size-fits all model, strategies must be adaptive to local context and balance the competing priorities of multiple ecologies. However, as our structural economic analysis indicates, the significant concentration of corporate power within both broiler and layer production, as well as at the retail level, means that major corporations will act as key gatekeepers to any such change. Commenting on resilience in the poultry sector, the NFU, highlighted planning regulations as a top priority for the industry, while noting that ‘changes to production systems and breeds should be evidence based and/or consumer led with fair returns on investment’ (NFU 2024c). This latter point starkly exposes the socio-economic-ecological tension at the heart of food system transformation, with the recent high profile withdrawals of major companies from the BCC further underscoring the inherent fragility of system change.

Beyond these considerations, however, lie more challenging debates around whether the current level of intensive production and low-cost poultry has long-term viability, particularly regarding ecological sustainability. In recent times there have been numerous calls for significant reductions in national meat consumption in Britain (Willett *et al* 2019, CCC 2020, McCulloch 2026). Alternative poultry futures could include those with lower levels of national meat consumption and where other models of production make significant contributions, rather than those which offshore production and increase reliance on cheap imported poultry.

## 4 Conclusion

The recent outbreaks of HPAI in GB illuminate the challenges of prevailing biosecurity regimes and on farm-level interventions in securing poultry production systems. Through this paper, we have examined how the structure of poultry production intersects with uneven geographies of production and risk, spatial planning challenges, and biosecurity concerns.

We argue that biosecurity must be conceptualised as a dynamic and situated set of negotiations, operating across space and time to reduce the vulnerabilities of food production. As the ongoing outbreaks since 2021 have shown, the resilience of GB’s poultry needs to be strengthened by moving away from reliance on farm-level interventions and movement restrictions; instead, it must be understood as a product of interdependent ecologies; biological, social, economic, and political. Within this framework, poultry resilience is co-produced by the structure of national and international agri-food markets, leisure based rural activities, the legacies of planning decisions, the response of industrial infrastructure to social movements, and the often-uneven capacities of producers to respond to risk.

This framing calls for a long-term, macro-level approach to security and resilience that addresses not only the immediacy of pathogen control but also the structural conditions that shape vulnerability in the first place. Doing so requires looking beyond the farm as the primary unit of intervention and embracing a multi-faceted approach which considers ecological flows, migratory bird pathways, social and economic land use, environmental considerations, and questions of sustainable protein production and consumption. Future growth, and transformation, of the poultry sectors must consider these multiple dimensions to reduce inherent vulnerability, and improve the security and resilience of production.

## Figures and Tables

**Table 1:** Summary of in-depth interviews across GB’s poultry sectors.

Sector	Firms	Industry organisations	Other	
Production & distribution				
Poultry meat	5	1	Veterinarians	5
Eggs	2	1	Training	2
Game birds	1		Insurance	1
Retail	1			

**Table 2:** Summary of key terms.

Term	Definition
Production type:	
Barn	Birds raised in a large barn where they can move around. Broiler stocking density of up to 17 birds per square metre or 38 kg m^−2^ Layer stocking density of up to 9 hens per square metre.
Enriched (colony) cage	Cage systems used to rear layer chickens up to 13 birds per square metre. Cages can hold up to 90 birds per cage.
Free range	A barn system with outdoor access. Outdoors, birds have access to 4 square metres per bird.
Organic production	A barn system with lower indoor stocking density (6 birds per square metre), outdoor access, and additional requirements for types of inputs used during production. Outdoors, birds have access to 10 square metres per bird.
Poultry terms:	
Broiler	A chicken raised for meat
Pullet	A young layer chicken 16 weeks old or less.
Better chicken commitment	Voluntary scheme to improve welfare of broilers Maximum stocking density of 30 kg m^−2^ Breeds meeting RSPCA broiler breed welfare assessment protocol criteria Additional environmental standards (e.g. over 50 lux of light, perches)
Cage free 2025	Voluntary scheme for egg production to move away from the use of enriched (colony) cages

**Table 3:** Comparative contribution of layer systems to national egg production.

Country	Enriched colony cages (%)	Barn (%)	Free-range & organic (%)	Source
UK	21	7	72	(British Lion 2024)
France	55	23	22	(Cocotine 2020)
Germany	4.3	58	37.7	(Negrici 2024)
Poland	71.8	21.3	6.9	(CIWF 2022)
Netherlands	10	80	10	(O’Keefe 2012)

**Table 4:** Shooting season for game in Britain (BASC 2026).

Species	Season
Pheasant	October 1st—February 1st
Partridge	September 1st—February 1st
Ducks	September 1st—January 31st

## Data Availability

Restrictions apply. Data cannot be shared publicly via a URL/DOI. Due to the sensitivity of data collected through in-depth interviews, data is not publicly available. Requests for information can be made by contacting the Royal veterinary College’s Publications Repository (contact via PublicationsRepos@rvc.ac.uk), for researchers who meet the criteria for access to confidential data. Supplementary data 1 available at: https://doi.org/10.1088/2976-601X/ae8dd4/data1.

## Footnotes

4 Great Britain refers to the combined nations of England, Scotland, and Wales.

5 Avian influenza reporting seasons run from the 1st October to the 30th September

6 The United Kingdom refers to region of Great Britain and Northern Ireland.

7 A stakeholder workshop was held in January 2026 in Cambridge attended by 25 stakeholders from various poultry industries and organizations, government, and academics.

8 Assuming the following: Broiler live weight at slaughter 2.2 kg. Layer bird live weight 2 kg. Annual cumulative egg mass of laying hens of 15 kg. Pheasant, partridge, mallard duck for shooting weight of 1.2 kg, 0.5 kg, 1.2 kg respectively. Turkey, duck, and goose for retail consumption weight of 6 kg, 2.2 kg, and 4.5 kg respectively.

9 Achieving 3.49 kg in 63 d (www.hubbardbreeders.com/newsroom/hubbard-press-release/1000-hubbard-introduces-redbro-for-the-bcc-market.html).

10 Formally the joint venture company Hook2Sisters, which was acquired by Ranjit Singh and Boparan Private Office in August 2025.

11 Formally Moy Park.

12 Interview data; 241122_1742 poultry organisation representative.

13 Interview data; 241122_1742 poultry organisation representative.

14 Interview data; 241104_2144 poultry firm employee.

15 Interview data; 250130_1000 poultry veterinarian.

16 In 1998, a junior health minister falsely said that most eggs were infected with Salmonella, resulting in an immediate 60% reduction in eggs sales and the slaughter of four million hens and 400 million unwanted eggs (Poultry Site 2006, Randall 2012).

17 Though modern free-range systems are arguably vastly different to pre-war free-range production.

18 Interview data; 250214_0900 government veterinarian.

19 Interview data; 241213_1400 egg producer.

20 A similar 12-week limit for free-range broiler birds has also now been removed (Defra 2025d, Defra 2025d).

21 Interview data; 241128_2043 insurance provider.

22 Interview data; 250124_1400 industry representative.

23 Interview data, 250110_1400, poultry veterinarian.

24 The political and economic union between Belgium, the Netherlands, and Luxembourg.

25 The cage free 2025 commitment for the layer sector and the reduced stocking density for broiler production.

26 Interview data; 250110_1400 poultry veterinarian.

27 For example, West Berkshire Council rejected a planning decision of Sutton Estates to demolish six existing poultry sheds to build a new unit to house 32 000 chickens (Waple and Hinman 2025).

28 After an appeal by the campaign group River Action, the high court in Cardiff overturned a planning permission for a ‘poultry mega-farm’ in Shropshire due to a ruling that ‘the council had failed to lawfully assess the impacts of spreading manure or digestate’ (Laville 2025b).

29 Interview data; 241217_0900 broiler firm employee.

30 Interview data, 241213_1400 layer firm employee.

31 Interview data, 241128_2043 insurance provider

32 An estimate of this is made by one of the APHA veterinarians who has to visually inspect each poultry house and decide the proportion of birds which are alive and not showing clinical signs of HPAI.

33 Interview data, 241120_1400 private poultry veterinarian.

34 For the Pig Industry foot and mouth disease scheme, the government contributed 80% of the levy fund and industry 20%.

35 Interview data; 250130_1100 private poultry veterinarian.
